# Subclinical Ketosis on Dairy Cows in Transition Period in Farms with Contrasting Butyric Acid Contents in Silages

**DOI:** 10.1155/2014/279614

**Published:** 2014-11-25

**Authors:** Fernando Vicente, María Luisa Rodríguez, Adela Martínez-Fernández, Ana Soldado, Alejandro Argamentería, Mario Peláez, Begoña de la Roza-Delgado

**Affiliations:** ^1^Servicio Regional de Investigación y Desarrollo Agroalimentario (SERIDA), 33300 Villaviciosa, Asturias, Spain; ^2^Sociedad Asturiana de Servicios Agropecuarios, SL (ASA), 33199 Granda, Siero, Asturias, Spain

## Abstract

This study examines the relationship between subclinical ketosis (SCK) in dairy cows and the butyric acid content of the silage used in their feeding. Twenty commercial farms were monitored over a period of 12 months. The feed at each farm and the silages used in its ration were sampled monthly for proximal analysis and for volatile fatty acid analysis. A total of 2857 urine samples were taken from 1112 cows to examine the ketonuria from about 30 days prepartum to 100 postpartum. Wide variation was recorded in the quality of silages used in the preparation of diets. Approximately 80% of the urine samples analyzed had no detectable ketone bodies, 16% returned values indicative of slight SCK, and the remainder, 4%, showed symptoms of ketosis. Most of the cases of hyperkenuria were associated with the butyric acid content of the silage used (*r* ^2^ = 0.56; *P* < 0.05). As the metabolizable energy content of the feed was similar, no relationship was observed between the proportion of cows with SCK and the energy content of the feed. In our study, the probability of dairy cows suffering SCK is higher when they are eating feed made from silage with a high butyric acid content (35.2 g/kg DM intake).

## 1. Introduction

Subclinical ketosis (SCK) in dairy cows is a common metabolic disorder that can appear during the transition period, dry period, or calving, or in early lactation (Duffield et al. [1]), where the highest incidence of SCK occurs within the first 2 to 3 weeks of lactation [2]. The disorder is characterized by a high concentration of circulating ketone bodies (acetone, acetoacetate, and *β*-hydroxybutyrate) in the absence of any clinical signs of ketosis [3]. It is a strong determinant of the health and performance of cows throughout lactation and causes economic losses due to reduced milk production [4] and is associated with the occurrence of diseases such as displaced abomasum [5] and with impaired fertility [6].

The SCK is caused by a negative energy balance in high-producing dairy cows. Milk production increases dramatically after calving when constraints on dry matter intake force dairy cows to mobilize body fat to meet their energy requirements. This mobilized overload of fat is metabolized by the liver and is converted into ketone bodies. In addition, persistent ketosis problems have been associated with feeding cows' ketogenic silage [7]. During forage ensiling, lactic acid fermentation is desirable, as well as acetic acid fermentation only in a small amount, but undesirable butyric and alcoholic fermentations may also occur. Grass silage that is chopped too wet, or that is low in water-soluble carbohydrates, promotes the growth of* Clostridium* spp. These ferment some carbohydrates to butyric acid instead of the desired lactic acid [8]. When animals consume silage, this butyric acid is metabolized to ketone bodies [9].

Butyric fermentation is common in grass silage in the wet-temperate areas [10]. The eventual appearance of SCK in cows on these areas might depend on the amount of butyric acid consumed and on the presence of other risk factors for ketosis (early lactation, high production, low dietary energy, etc.). The aim of the present paper was to examine the relationship of SCK in dairy cows in the transition period in herds with contrasting butyric acid content in the silages used in preparing their ration.

## 2. Materials and Methods

Twenty commercial dairy farms in northern Spain (Principality of Asturias) making up 1112 cows, approximately 1.25% of all dairy cows of the region, were examined over a period of 12 months. Principality of Asturias is a wet-temperate area located in Atlantic coast and has a marine west coast climate, with warm and wet summers and fairly mild winters. According to the farm record databases, the average milk production per cow was 9100 ± 790 kg, with 3.73 ± 0.22% of fat and 3.13 ± 0.13% of protein. The farms were visited monthly and urine samples were taken from cows at risk of developing ketosis: from about 30 days prepartum to 90 days postpartum, a total of 2831 urine samples were taken. Urine was sampled directly from the bladder by urethral catheter and the ketone body concentration determined using Combur-test strips (F. Hoffmann-La Roche Ltd, Basel, Switzerland). The semiquantitative results were expressed in terms of four ketosis categories: trace (<5 mg AcAc/dL), low (5 up to 50 mg/dL), moderate (50 up to 150 mg/dL), and high (>150 mg/dL). Low and moderate levels of AcAc mean subclinical ketosis and high level means clinical ketosis. All work was conducted according to the standard of European Union Animal Welfare Directive Number 86/609/EEC.

All rations were formulated according to animal requirements with different feedstuffs. Grass and maize silage were included in the diet of 90% of the farms, ryegrass silage in 50% of the farms, alfalfa or grass hay in 40% of the farms, and mixed concentrate feeds in 100% of the farms. When the maize silage was included in the diet, the average content (on dry matter basis) was 33.6% (range: 18 to 68%). Grass silage was included averaging 28.7% (range: 10 to 56%) and ryegrass silage with an average of 19.2% (range: 9 to 35%). The proportion of concentrate in all diets was between 21 and 26% on dry matter basis. The rations were sampled on the same day as urine sampling, as well as the silage sampled that was used to make rations. Feed samples were dried at 60°C for 24 h and then ground through a 0.75 mm mesh. The dry matter (DM), ash, crude protein (CP), and neutral detergent fibre (NDF) and acid detergent fibre (ADF) in rations and silages and starch in rations and maize silages were determined by near infrared spectroscopy (NIRS) in reflectance mode using a FOSS NIRSystems 5000 instrument (FOSS NIRSystems, Inc., Laurel, MD, USA). Silage juice was extracted using a piston press before drying, and volatile fatty acid (VFA) and lactic acid concentrations were determined by HPLC (Waters Alliance, Milford, MA, USA).

A linear regression analysis was done to establish the relationship between butyric acid concentration and the four categories of ketonuria, including the farm as random factor, in order to demonstrate the relationship between butyric acid concentration and the appearance of subclinical and clinical ketosis [11].

## 3. Results

Data based on farm records and survey responses from a questionnaire to farmers showed that the diet offered by cow was 23.3 ± 1.25 kg DM per day. The average chemical composition of diet and silages is showed in Table 1. Wide variation was recorded between the silages used in the diets, but the final rations had similar nutrient contents and fermentative values. The silages were generally well-made, especially maize silage, with negligible levels of butyric acid. The grass and ryegrass silages used were characterized by high levels of butyric acid (26 and 15 g/kg DM, resp.) and showing a wide range of butyric concentration (0 to 61.3 g/kg DM for grass silage and 0 to 38.3 g/kg DM for ryegrass silage). However, there was not observed any farm that includes poorly fermented silage routinely in the diets.

**Figure 1:**
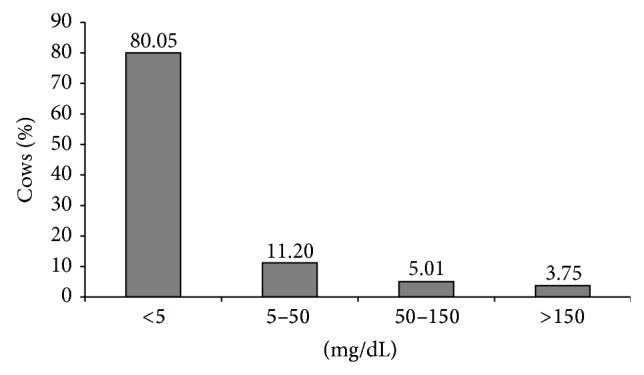
Distribution frequency of cows according to ketone bodies content in urine (mg AcAc/dL): <5 mg/dL: trace; 5–50 mg/dL: low; 50–150 mg/dL: moderate; (subclinical ketosis: low + moderate); >150 mg/dL: high (clinical ketosis).

**Figure 2:**
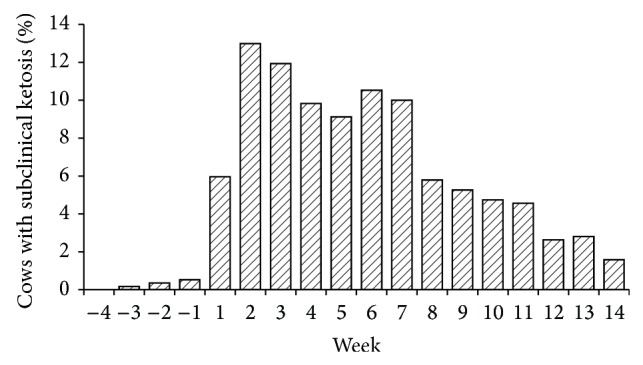
Distribution frequency of subclinical ketosis status (low and moderate ketone bodies excretion: 5–150 mg AcAc/dL) according to week postpartum.

The distribution of subclinical ketosis was similar between farms, without observing any herd with a significant concentration of cases of subclinical or clinical ketosis.  Figure 1 shows the distribution frequency of ketone bodies in urine in the cows studied. Few postpartum cases of subclinical and clinical ketosis were observed. About 80% of all the urine samples analyzed had no detectable ketone bodies and 16% provide values low and moderate of ketone bodies, indicative of SCK, and less than 4% of the samples show values compatible with clinical ketosis. The proportion of cows with SCK (low and moderate level of AcAc in urine) is illustrated in Figure 2, showing that prevalence was higher from the second until the seventh week postpartum, falling afterwards progressively.

**Figure 3:**
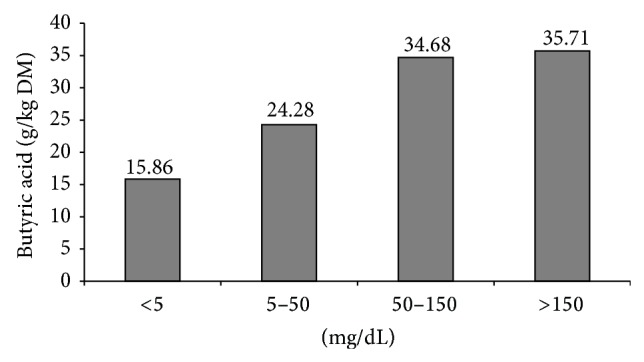
Estimated butyric acid intake in each urine distribution ketosis status: trace, <5 mg AcAc/dL; low, 5–50 mg AcAc/dL; moderate, 50–150 mg AcAc/dL; high, >150 mg AcA/dL.

Figure 3 shows the estimated butyric acid intake associated with the different descriptors (trace, low, moderate, and high) of urine ketone body concentration. Cows with ketosis problems (either SCK or clinical ketosis) consumed feed with the highest butyric acid concentrations, especially with moderate and high ketonuria. In all cases, the metabolizable energy content of the feed was similar (10.84 MJ ME/kg DM); therefore there does not seem to be a relationship between the proportion of cows with SCK, or clinical ketosis, and the energy content of ration. There was, however, a positive relationship observed between the concentration of ketone bodies in the urine and the butyric acid content of the silage used in the ration (*r* ^2^ = 0.56; *P* < 0.05).

## 4. Discussion

Diagnosing ketosis in large animal populations requires a different approach (urine testing for ketone bodies) to that used when diagnosing ketosis in an individual animal (blood testing for *β*-hydroxybutyric acid). In the present work, cow-side urine ketosis testing was used since it is more economic than blood *β*-hydroxybutyric acid testing and provides immediate results. Urine ketone tests have a reputation for poor specificity, but recent data suggest that their specificity and sensitivity are good compared to the blood *β*-hydroxybutyric acid test [12].

A strongly negative energy balance due to high milk production is one of the most important factors influencing the appearance of SCK between three and six weeks after calving. The results of the present work showed an average prevalence of SCK in the two first months postpartum of 9.2%, which is in agreement with the lower limit of the range (8.9 to 34%) reported by [2]. Further, the peak of prevalence between the second and seventh week of lactation is in agreement with that reported by [13].

High concentrations of butyric acid have traditionally been associated with anaerobically unstable silages made from crops with high buffering capacities, such as grass [9]. This leads to an insufficient decline in the pH during the primary fermentation phase [14]. Butyric fermentation can be prevented by inhibiting the detrimental effect of oxygen penetration into the silage via the addition of an inhibitor of aerobic deterioration, such as* Lactobacillus buchneri*, or by wilting the forage before ensiling [15]. Cows can metabolize the butyrate produced by ruminal fermentation (about 750 g/day), but additional dietary butyric acid increases the risk of ketosis. About 75% of the additional ruminal butyrate is converted to blood *β*-hydroxybutyrate and causes ketosis [16]. Similarly, Lingaas and Tveit [17] reported that daily doses of 50 to 100 g of butyric acid can cause ketosis, and that doses of over 200 g can induce severe ketosis. About 450 to 950 g of butyric acid will reliably induce severe ketosis in nearly any cow in early lactation [18]. Tveit et al. [7] reported that a concentration of 18.5 g/kg DM of butyric acid in silage can induce severe ketosis. In the present study, grass silage had a butyric acid concentration (25.5 g/kg DM) higher than the mean. Grass silages have long been considered linked to SCK since they contain much higher* Clostridium* spp. spore concentrations than corn or ryegrass silage [9]. Ryegrass silage is more resistant to clostridial fermentation because of its abundance in water-soluble carbohydrates. Maize silage rarely supports clostridial growth since it contains high carbohydrate reserves that can be transformed into lactic acid during the first days of fermentation, helping pH to fall below 4 [8]. Therefore, silage butyric acid levels should be kept low and monitored frequently. Small differences in the content of butyric acid in silage could induce high differences in the SCK cases in transition cows. However, the potential number of cases of SCK could be reduced by the use of maize silage, which would have provided an energy supplement.

## 5. Conclusions

On dairy farms in northern Spain, the probability of dairy cows suffering SCK is higher when they are provided feed made from silage with high butyric acid content. Farmers would be well advised to produce good quality silage, that is, low in butyric acid, in order to reduce the possibility of cows in transition developing SCK. The levels of butyric acid in silage should be carefully monitored.

## Figures and Tables

**Table 1:** Average chemical composition and standard deviation of diets and silages and fermentative characteristics of silages.

Variable	Diets	Maize silage	Grass silage	Ryegrass silage
*n*	93	54	63	22
Dry matter (g/kg fresh matter)	521.5 ± 12.53	344.4 ± 4.99	350.4 ± 15.45	302.3 ± 13.54
Organic matter (g/kg DM)	920.2 ± 1.24	963.3 ± 1.08	881.2 ± 3.91	882.4 ± 7.80
Crude protein (g/kg DM)	155.7 ± 1.67	78.5 ± 0.97	112.3 ± 3.41	108.2 ± 9.80
Neutral detergent fibre (g/kg DM)	347.5 ± 11.58	438.8 ± 10.09	580.9 ± 5.84	541.0 ± 11.37
Acid detergent fibre (g/kg DM)	234.2 ± 7.71	255.8 ± 6.84	350.5 ± 3.10	331.9 ± 8.79
Starch (g/kg DM)	223.8 ± 4.27	347.2 ± 3.06	nd	nd
Metabolizable energy (MJ/kg DM)	10.8 ± 0.24	11.1 ± 0.01	9.9 ± 0.04	9.8 ± 0.09
pH	nd	3.8 ± 0.05	4.6 ± 0.08	4.3 ± 0.09
Lactic acid (g/kg DM)	nd	40.3 ± 2.00	32.7 ± 3.20	47.0 ± 4.31
Acetic acid (g/kg DM)	nd	15.0 ± 1.46	10.9 ± 1.24	10.5 ± 1.65
Butyric acid (g/kg DM)	nd	1.2 ± 0.50	25.5 ± 3.00	15.4 ± 2.42
Propionic acid (g/kg DM)	nd	0.3 ± 0.17	1.0 ± 0.31	1.4 ± 0.39

nd: not determined.
